# Using Community Engagement to Create a Telecoaching Intervention to Improve Self-Management in Adolescents and Young Adults With Cystic Fibrosis: Qualitative Study

**DOI:** 10.2196/49941

**Published:** 2025-01-20

**Authors:** Christina L Duncan, Emily F Muther, Jennifer J Lindwall, Kristine Durkin, Elizabeth Ruvalcaba, Eliza Williamson, Corrine Ahrabi-Nejad, Evelyn Bord, Angela Green, Megan L Harrison, Deepika Polineni

**Affiliations:** 1 Department of Psychology Oklahoma State University Stillwater, OK United States; 2 Department of Psychiatry University of Colorado School of Medicine Aurora, CO United States; 3 Department of Psychiatry and Human Behavior Warren Alpert Medical School of Brown University Providence, RI United States; 4 Division of Pulmonary and Critical Care Medicine Department of Medicine Johns Hopkins School of Medicine Baltimore, MD United States; 5 National Institute of Children's Health Quality Boston, MA United States; 6 Division of Pediatric Hematology, Oncology, and Stem Cell Transplantation Columbia University Medical Center New York, NY United States; 7 Division of Respiratory Diseases Boston Children's Hospital Boston, MA United States; 8 Research and Sponsored Projects Administration Children's Mercy Hospital Kansas City, MO United States; 9 Division of Allergy & Pulmonary Medicine Washington University School of Medicine St Louis, MO United States

**Keywords:** cystic fibrosis, telecoaching, self-management, community engagement, community partner, intervention development

## Abstract

**Background:**

Adolescents and young adults (AYA) with cystic fibrosis (CF) are at risk for deviating from their daily treatment regimen due to significant time burden, complicated daily therapies, and life stressors. Developing patient-centric, effective, engaging, and practical behavioral interventions is vital to help sustain therapeutically meaningful self-management.

**Objective:**

This study aimed to devise and refine a patient-centered telecoaching intervention to foster self-management in AYA with CF using a combination of intervention development approaches, including an evidence- and theory-based approach (ie, applying existing theories and research evidence for behavior change) and a target population–centered approach (ie, intervention refinement based on the perspectives and actions of those individuals who will use it).

**Methods:**

AYA with CF, their caregivers, and health professionals from their CF care teams were recruited to take part in focus groups (or individual qualitative interviews) through a video call interface to (1) obtain perspectives on the overall structure and logistics of the intervention (ie, Step 1) and (2) refine the overall framework of the intervention and obtain feedback on feasibility, content, materials, and coach training (ie, Step 2). Qualitative data were analyzed using a reflexive thematic analysis process. Results were used to create and then modify the intervention structure and content in response to community partner input.

**Results:**

For Step 1, a total of 31 AYA and 20 clinicians took part in focus groups or interviews, resulting in 2 broad themes: (1) video call experience and (2) logistics and content of intervention. For Step 2, a total of 22 AYA, 18 clinicians, and 11 caregivers completed focus groups or interviews, yielding 3 major themes: (1) intervention structure, (2) intervention materials, and (3) session-specific feedback. Our Step 1 qualitative findings helped inform the structure (eg, telecoaching session frequency and duration) and approach of the telecoaching intervention. Step 2 qualitative results generally suggested that community partners perceived the feasibility and practicality of the proposed telecoaching intervention in promoting self-management in the face of complex treatment regimens. Extensive specific feedback was used to refine our telecoaching intervention before its efficacy testing in subsequent research. The diverse community partner input was critical in optimizing and tailoring our telecoaching intervention.

**Conclusions:**

This study documents the methods and results for engaging key community partners in creating an evidence-based behavioral intervention to promote self-management in AYA with CF. Incorporating the lived experiences and perspectives of community partners is essential when devising tailored and patient-centered interventions.

## Introduction

Cystic fibrosis (CF) is a progressive genetic disorder that impacts many systems in the body, including potentially causing chronic lung infections, gastrointestinal abnormalities that create malabsorption and make it difficult to grow and gain weight [1], impairment of sexual health and reproduction [2,3], and numerous other comorbidities [4]. CF is estimated to affect approximately 40,000 children and adults in the United States and about 105,000 people worldwide [5,6]. Historically, children with CF rarely lived to adulthood. Currently, however, the median expected survival age of a child born with CF in 2023 in the United States is 68 years [7]. Recent improvement in survival is primarily due to the advances in therapeutics, that is, cystic fibrosis transmembrane conductance regulator (CFTR) modulators, or CFTR corrector and potentiator medications, which ameliorate pulmonary disease [8]. Still, the potential to benefit from these new therapeutics is paralleled by the increasing complexity and time required to complete multiple daily treatments.

Adolescents and young adults (AYA) with CF are at particular risk for nonadherence to their treatment regimen, given stressors common to this developmental period, including social pressures and increased academic or work demands [9]. Furthermore, people with CF report a significant time burden (ie, more than 1 hour) in completing their daily therapies [10]. It is not surprising, then, that adherence to prescribed treatment regimens is a common problem in CF, with adherence rates to all CF treatments ranging from 35% to 75%, while CF medication-specific adherence spans 31% to 79% [11-13]. This wide range in adherence rates stems from variability in measurement approach (ie, self-report vs objective measures), age of the individual, differences across treatment components, and other factors [14]. People with CF are unable to benefit from cutting-edge medications and interventions if barriers exist that prevent therapeutically meaningful self-management. As treatments in CF expand to include the groundbreaking use of CFTR modulators, efforts to improve medication and treatment self-management are of paramount importance. Identifying and developing effective behavioral interventions that are patient-centered, engaging, and practical (for both people with CF and care teams) will be critical to successful implementation and subsequent positive impact in helping individuals follow their CF treatment.

Although telecoaching has been used to successfully manage other health conditions [15,16], it has not been adopted to address self-management in people with CF. The flexibility of telecoaching affords the opportunity to take an accessible and patient-centered approach to identify individualized self-management concerns and address them with relevant, efficacious interventions. Indeed, a range of behavioral interventions have been effective or promising in addressing self-management in patients across disease populations [15,17,18]. These interventions include organizational and behavioral strategies, problem-solving around barriers to self-management, motivational interviewing, and educational approaches [19]. Core aspects of these interventions can be woven into brief telecoaching sessions, especially if these strategies are linked specifically to the personal barriers that patients report facing with their daily regimen. In addition, given that fewer outpatient visits and poor follow-up by providers negatively impact self-management [20], brief telecoaching sessions with a trusted and personally known health care clinician offer a pragmatic and accessible way to link clinicians and patients on a more regular basis. Yet, little is known about its clinical effectiveness in improving self-management in people with CF.

The goal of this study was to obtain and apply community partner feedback to develop (Step 1) and refine (Step 2) a novel and patient-tailored telecoaching intervention to enhance self-management in adolescents and young adults with CF (ages 14-25 years). In our subsequent line of research, the telecoaching intervention will be tested for its feasibility, acceptability, and effectiveness. Our ultimate goal is to establish an accessible, acceptable, and efficacious telecoaching intervention to offer during routine care across CF care centers in the future.

## Methods

### Study Design

**Figure 1:**
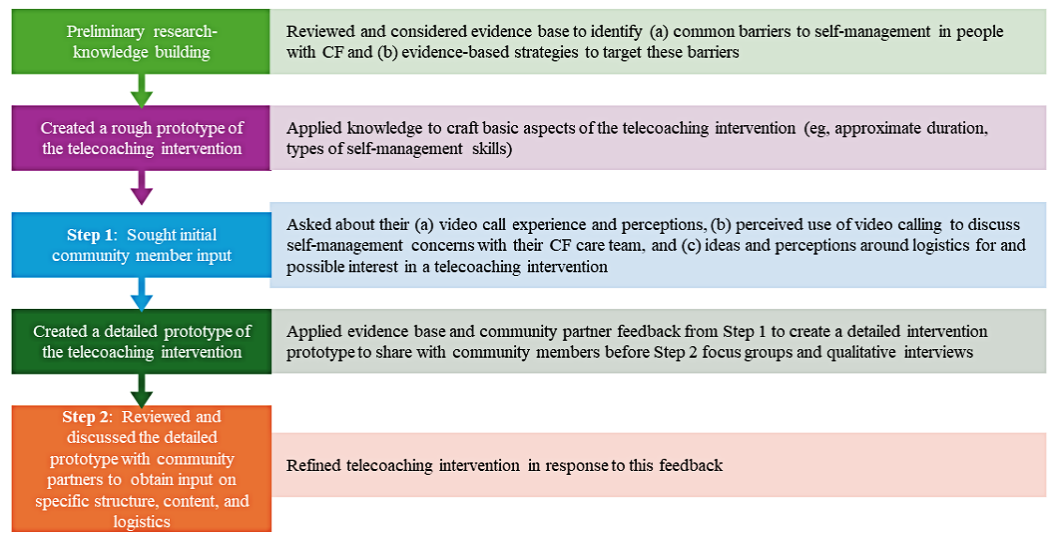
Study design. CF: cystic fibrosis.

Figure 1 shows the study design, which consisted of a combination of intervention development approaches, including an evidence and theory-based approach (ie, applying existing theories, like social cognitive theory [21], and research evidence for health behavior change) and a target population-centered approach (ie, intervention refinement based on the perspectives and actions of those individuals who will use it [22]). Consistent with guidance from O’Cathain et al [22], Step 1 pertained to key aspects of intervention development, whereas Step 2 focused on intervention design.

### Sample

Participants included AYA with CF (ie, “patients”), their caregivers, and health care professionals (ie, “clinicians”) from their CF care teams. From November 2017 to June 2018, research staff recruited participants from 5 CF centers in the United States (Children’s Hospital Colorado, National Jewish Health, Northwestern University, University of Kansas Medical Center, and West Virginia University). Together, these CF centers provided a diverse population from which to draw our sample. Eligible patients were recruited during routine clinic visits and were English-speaking, aged 14 to 25 years, diagnosed with CF, and prescribed at least one respiratory medication (eg, inhaled antibiotic, dornase alfa, hypertonic saline, oral azithromycin, ivacaftor, lumacaftor, and ivacaftor combination), used a vest device with usage monitor (ie, SmartVest [Electromed Inc], Hill-Rom [Baxter International], Afflovest [Rotech Healthcare], or Respirtech [Koninklijke Philips]) for airway clearance, and had access to a device with an internet connection to host a teleconference meeting. Patients were not eligible if they had a history of lung transplant. English-speaking primary caregivers who resided with a patient participant, (and who received permission to participate from a patient who was 18 years or older) were recruited too. Eligible CF care clinicians were English-speaking and employed within a participating accredited Cystic Fibrosis Foundation care center; study staff recruited them to take part in this research.

### Study Procedures

Before Step 1, the study team devised a rough prototype of the telecoaching intervention. Step 1 of intervention development involved conducting community partner interviews (February-August 2018), using a semistructured guide, to obtain perspectives and thoughts on the overall intervention structure and logistics—that is, access to the internet and smart devices, experience and perspectives using video calling in general, experience with and potential application of video calling to communicate with the patients or CF care team and the potential application of video calling to the discussion of self-management concerns, preferences for who serves as a coach, some overall intervention feasibility (eg, frequency of sessions) questions, and potential interest in this type of intervention. The study team met to discuss the interview information needed to fully create the intervention prototype (eg, access to the internet, video calling experience, and interest). The first author created the initial draft of the interview guide, which was then jointly edited by the study team. The interview guide generally covered the same topics across informants (more details in Multimedia Appendix 1).

Then, before Step 2, the study team expanded the creation of the telecoaching intervention, using findings from Step 1 and applying the research evidence base regarding specific, efficacious behavioral strategies (eg, problem-solving and behavioral activation) to target various common barriers that people with CF experience when managing their treatments. A detailed overview document of the proposed telecoaching intervention was shared with participants just before the Step 2 focus group or qualitative interviews, which took place from November 2018 to February 2019. This summary was used as a reference during the interviews, with its content reviewed and discussed. The interview guide again was created by the first author and subsequently edited by the study team, with the goal of obtaining specific feedback from community partners to refine the details of the telecoaching intervention structure, logistics, and content (more details in Multimedia Appendix 2).

In addition to AYA with CF and their health care clinicians, caregivers of enrolled AYA with CF also engaged in Step 2 interviews. For patients and clinicians, the overview document included key points (eg, session duration, coach professions, and basic structure), a description of what skill sessions were, sample session activities, an overall intervention timeline and flow of sessions, and a sample intervention timeline and session flow for a hypothetical participant. The caregiver overview handout was a 2-page intervention summary (as caregivers were not expected to be participants in the intervention). All informants were asked to comment on the overall structure and duration of the telecoaching program; feedback on specific skill sessions, intervention materials, and their format (paper vs digital); and feasibility and preference for session timing (eg, work hours, nights, weekends). Clinicians were also asked what training the coaches might need, and caregivers were asked to share any caregiver-specific considerations the team should keep in mind.

### Research Team and Reflexivity

Research staff (ER, EW, KD, CA-N, and MH) carried out the interviews and coding. These individuals were research staff, with KD, CA-N, and MH working in the labs of the lead investigators (CLD and DP). All were trained and experienced in conducting interviews. Although none of the interviewers had previous relationships with the participants, KD and CA-N were advanced doctoral clinical psychology students who had supervised experience in clinical interviewing, including building rapport. At the outset of all interviews, the interviewer introduced themselves, explained the purpose of the research, and began the meeting with an icebreaker activity. The study team was also comprised of 3 licensed and academic clinical psychologists (CLD, EFM, and JL), all with extensive clinical and research experience with people with CF. This experience, coupled with that of a pulmonologist fully dedicated to CF care (DP), provided combined strengths when discussing interpretations of data. Contributions from advanced research staff (EB and AG) ensured proper study management and data integrity, which helped reduce bias and enhance the reliability of our findings. Our entire study team was female; two of our members identified as people of color, and one as Hispanic.

### Qualitative Analysis

All interviews were conducted with an experienced coauthor interviewer (ER for Step 1 and EW for Step 2) using a video-conferencing platform. Adolescents (ages <18 years) and young adults (ages 18-25) were interviewed separately. Note that an 18-year-old attending high school was assigned to the adolescent group rather than the young adult group. Clinicians were grouped based on scheduling availability; thus, each focus group had a mix of professionals. Caregivers were grouped separately, depending on whether they were parents of an adolescent or young adult (as per patient cohort grouping above). All participants were encouraged to take part in a focus group; however, individual qualitative interviews (using the same guide) were offered to those not interested in a group format or to those with scheduling constraints. All groups had 1 interviewer, plus 1 staff member behind the scenes to address any potential technology concerns and to take notes. All focus groups and individual qualitative interviews were audio-recorded and transcribed by a paid service. Transcripts were cleansed by contrasting their content with the original recordings. All information also was deidentified.

Thematic analysis was performed for each informant group in an iterative manner using NVivo software (Lumivero) [23]. Experienced qualitative coders (ER, KD, and EW for Step 1; CA-N and MH for Step 2) conducted this analysis as data were obtained. A clear audit trail of notes and decision-making was established with files stored in a secure, shared account. Interviews for Steps 1 and 2 were conducted until saturation of themes was achieved upon iterative review of transcripts.

For both steps, the first author and 2 coders (primary and secondary) read the first transcript of each cohort, recording initial codes using the comment function in Microsoft Word. They discussed and established the initial coding frame and codebook. Then, the primary coder continued coding transcripts, while the secondary coder coded a random sample of each cohort of transcripts until at least 20% of transcripts were double-coded [24]. Initial kappa values between coders ranged from κ=0.61 to κ=0.73, indicating substantial agreement [25]. Throughout this process, discrepancies were discussed, and modifications to the codebook were made, as needed, in an iterative manner. Saturation (ie, no new themes arising) was attained in coding data for both steps. After coding was complete for all cohorts, the first author and 2 coders collaborated to organize the codes into a thematic structure.

After reflexive thematic analysis was complete for Step 1, the study team discussed all findings, considering different participant perspectives, and collectively made decisions regarding plans for creating the telecoaching intervention prototype before Step 2. In addition to the thematic analysis for Step 2, results were detailed in a Microsoft Excel table. This table consisted of the following columns: cohort (ie, patient, provider, and caregiver), target area (ie, intervention, coach training, and scheduling and logistics), specific topic (eg, general intervention, logistics, scheduling, and SMART goals session), relevant transcription excerpts, and action needed (ie, add, modify, and clarify). The study team carefully discussed each item until a decision was made regarding modifying the intervention. Information regarding each decision was recorded in 2 additional columns in the Excel file: (1) whether a change to the intervention prototype would be made based on the feedback (ie, yes or no) and study team response (a tracking system to record responsible parties and steps taken).

### Ethical Considerations

Study procedures were reviewed and approved by the Boston Children’s Hospital’s institutional review board (IRB-P00022531), which served as the Institutional Review Board of Record. Written informed consent was required from all participants (assent from minors, with parental consent). Potential participants were informed that they could opt out of the study, and it would not impact their standard CF care (patients and caregivers) or their standing within the CF care team (clinicians). All data were deidentified and coded with a unique participant number. Upon consenting to the study, patients and caregivers completed surveys as an Enrollment Assessment; each was compensated US $30. Clinicians completed a brief demographic survey upon enrollment, for which no compensation was provided. All participants were compensated US $30 for completing each qualitative interview.

## Results

### Step 1 Results

#### Participants

A total of 31 AYA patients with CF (13 adolescents; 18 young adults; more details in Table 1) participated across 9 focus groups (2-4 participants per focus group) and 10 one-on-one interviews. Focus groups lasted a mean of 59 minutes (SD 12; range 47-71), while individual interviews had a mean duration of 37 minutes (SD 13; range 29-61). A total of 20 clinicians (more details in Table 2) were interviewed across 6 groups (2-4 participants each), lasting 64 minutes on average (SD 6; range 51-68).

**Table 1:** Demographic and medical characteristics of participants (patients).

Patients			Overall (N=38)		Step 1 (N=31)		Step 2 (N=22)
Age, mean (SD)			19.8 (3.8)		19.8 (3.8)		19.9 (3.88)
Female, n (%)			22 (57.9)		20 (65)		16 (73)
White, non-Hispanic, n (%)			31 (81.6)		25 (81)		17 (77)
White, Hispanic, n (%)			4 (10.5)		4 (12.9)		4 (18.2)
Other, unspecified, n (%)			2 (5.3)		1 (3.2)		0 (0)
Other, Hispanic, n (%)			1 (2.6)		1 (3.2)		1 (4.5)
**Household income (US $), n (%)**							
	<60,000	6 (15.8)		3 (10)		2 (9)	
	60,000 to <120,000	7 (18.4)		6 (19)		4 (18)	
	≥120,000	7 (18.4)		6 (19)		4 (18)	
	Do not know or refuse to answer	18 (47.4)		16 (52)		12 (55)	
**Insurance, n (%)**							
	Private or military	32 (84.2)		26 (84)		19 (86)	
	Public or no insurance	6 (15.8)		5 (16)		3 (14)	
**FEV1^a^ percent predicted, mean (SD)**			79.8 (22.2)		82.8 (21)		84 (21)
	≥70%, n (%)	26 (68.4)		23 (74)		17 (77)	
	40-69%, n (%)	10 (26.3)		7 (23)		4 (18)	
	<40%, n (%)	2 (2.3)		1 (3)		1 (5)	
BMI percentile, mean (SD)			51.8 (24.6)		56.2 (23.2)		68.1 (10.7)
BMI, mean (SD)			23.2 (3.3)		23.5 (3.2)		23.1 (3.4)
*Pseudomonas aeruginosa*, n (%)			21 (55.3)		18 (58)		12 (54)
Gastroesophageal reflux disease (GERD), n (%)			16 (42.1)		12 (39)		9 (41)
Cystic fibrosis–related diabetes (CFRD), n (%)			15 (39.5)		12 (39)		9 (41)
Pancreatic insufficiency, n (%)			37 (97.4)		30 (97)		21 (95)
**F508del^b^, n (%)**							
	Homozygous	22 (57.9)		16 (52)		12 (55)	
	Heterozygous	15 (39.5)		14 (45)		10 (45)	
	Other	1 (2.6)		1 (3)		0 (0)	
Treatment complexity score [26], mean (SD)^c^			18.9 (5)		19 (5.5)		19 (5.8)

^a^Forced Expiratory Volume in one second.

^b^Delta F508 mutation, the most common genetic mutation in cystic fibrosis.

^c^Higher scores indicate a more complex regimen (range 0-76).

**Table 2:** Demographic and medical characteristics of participants (clinicians).

Clinicians		Step 1 (N=20)	Step 2 (N=18)
Female, n (%)		18 (90)	16 (89)
White, non-Hispanic, n (%)		20 (100)	18 (100)
**Clinician role, n (%)**			
	Nurse	2 (10)	2 (11)
	Nurse practitioner (advanced practice nurse)	2 (10)	2 (11)
	Nutritionist or dietitian	1 (5)	1 (6)
	Physical therapist	1 (5)	1 (6)
	Physician	2 (10)	1 (6)
	Psychologist or psychiatrist	1 (5)	1 (6)
	Registered nurse	3 (15)	3 (17)
	Respiratory therapist	4 (20)	3 (17)
	Social worker	4 (20)	4 (22)
**Clinical population, n (%)**			
	Adult	11 (55)	11 (61)
	Pediatric	4 (20)	3 (17)
	Both	5 (25)	4 (22)

#### Thematic Results

##### Overview

Results yielded two major themes: (1) video call experience and (2) logistics and content of the telecoaching intervention. Tables S1 and S2 in Multimedia Appendices 3 and 4 contain subthemes and descriptive quotes for these 2 themes, respectively. Step 1 thematic content is summarized below.

##### Video Call Experience

Patients’ previous use of video calling varied, with few reporting never having used video calls and the majority frequently using video calls for a range of purposes (eg, medical visits, personal communication with friends and family). Patients reported consistent availability of internet services and typically owned and had no restrictions on a personal device (ie, cell phone, laptop, or tablet). AYA differed somewhat on access, with adolescents having more restrictions (eg, parental settings). Patients identified benefits of video calling including the convenience, ease of use, infrequency of technical issues, ability to connect more with the other person, and their own comfort level. However, patients referenced some practical challenges (eg, video internet connectivity, privacy, and scheduling), as well as lack of motivation and changes in health, as possible concerns when using video calls for intervention delivery.

Clinicians perceived many benefits of conducting video calls with patients. They noted that video calling is convenient and allows for an alternative way to communicate with or reach patients. This method may be helpful to access previously hard-to-reach populations that live far away or have poor attendance to clinic visits. In addition, video calls could minimize missed school and workdays for patients and reduce concerns about infection control in clinics. Clinicians reported video calling allows them to gain new information as compared with discussing over the phone and allows them to see body language and reactions from patients. Video calling facilitates focus and reduces multitasking or distractions on the side of both patient and clinician. Finally, clinicians believed that patients may be more comfortable disclosing information because it is a less intimidating environment than a clinic.

Similarly, clinicians also reported some challenges in using video calls. They noted that patients may not have access to resources such as a device (phone or computer) or internet access to be able to engage in a video call in telecoaching. Access barriers may be financial or situational (eg, the situation at the time of call). Clinicians also reported the potential for issues with the platform itself and internet connection (eg, buffering or loss of connection), which can be distracting to or interrupt the conversation. Clinicians stated that video conferencing would require that both patients and clinicians receive additional training on how to use the platforms. Clinicians also expressed concerns for patient privacy (eg, challenging to find a private space to have the conversation) and felt that this might introduce an aspect of intrusiveness. Furthermore, they questioned whether video conferencing is an appropriate platform for conversations about mental health or other acute or sensitive issues. Concerns about difficulty scheduling calls and billing for services were expressed by many clinicians. Finally, clinicians wondered if video conferencing would impact rapport with patients and clinic attendance.

Regarding their perceptions of patient interest, many clinicians (17/20, 89%) stated they believed that patients would respond positively to the option for teleconferencing, particularly for convenience. They emphasized clinicians would need to be prepared that patients may be uncomfortable discussing self-management due to the calls feeling invasive or like a lecture instead of supportive. Clinicians had recommendations about subgroups of patients (eg, young, newly diagnosed, or parents) that they believed would benefit most from a telecoaching intervention.

##### Logistics and Content of Telecoaching Intervention

AYA with CF provided their suggestions about the qualifications of a coach for the proposed telecoaching intervention. Many patients confirmed they would be comfortable speaking with a coach about self-management concerns if the coach was knowledgeable about CF and they knew the person (ie, the coach was a member of their care team). When considering the profession of the coach, participants differed in their recommendations from a nurse, respiratory therapist, or social worker. AYA varied in their opinions of the frequency of video calls and length of the telecoaching intervention. The most common suggestion was that the duration of the intervention should be tailored to personal goals or needs. Other participants’ suggestions varied from a few months in length to 6 months to a year. Similarly, some patients with CF believed that the duration of telecoaching calls should vary based on situation and need, while others voiced that a duration of 30-60 minutes would suffice. AYA identified session topics (eg, mental health, changes in treatment regimen) they believed should be included in the intervention and those they thought were not appropriate for telecoaching (eg, sick visits or serious topics, such as surgery) and would require a face-to-face encounter.

While some clinicians recommended that session topics should be tailored to the patient’s goals and interests, others suggested a routine agenda for all video calls. They discussed that coaches should focus on emotionally sensitive issues (eg, mental health), identifying and addressing self-management barriers, and adjustment to life transitions (eg, moving to adult care or starting a job) during telecoaching intervention sessions. Several clinicians thought telecoaching would be useful for demonstrating a treatment technique or use of medical equipment. Many clinicians suggested the frequency of video calls should vary based on patient needs. Others voiced a specified frequency of calls (eg, every 1-2 weeks, monthly), more frequent sessions, or tapering sessions as potentially helpful and realistic for some patients. With respect to the length of intervention, many clinicians believed that 6 months was feasible, and the intervention needed to be a specified length for it to be effective. Few clinicians suggested the intervention should vary based on patient needs. Clinicians were mixed in their responses about how easy it would be for them to integrate telecoaching into their current practice. While many said they believe it would be feasible, others cited challenges around workload and scheduling (eg, time and space availability, fitting within the current workload). To integrate telecoaching calls, clinicians noted they would need support in how to allocate time around their own responsibilities and a patient’s schedule or activities and would need access to additional resources such as a private space and equipment. When discussing who on the CF care team should serve as a coach, some clinicians suggested a specific care team member (eg, nurse, social worker, respiratory therapist). However, clinicians reported that the coach chosen should depend on individual patient’s needs and existing relationships and therefore, identifying the coach may require a team approach. Clinicians suggested using visual or video tools to engage patients in telecoaching intervention sessions. Many clinicians suggested approaching patients with language other than “adherence” to preface intervention discussions as nonjudgmental.

### Step 2 Results

#### Participants

A total of 22 AYA (9 adolescents; 13 young adults), 18 clinicians, and 11 caregivers completed interviews. Table 3 shows the descriptive statistics for the AYA and clinician or caregiver cohorts, respectively. AYA participated in a total of 6 focus groups (2-4 participants each) and 5 individual interviews, lasting an average of 60 (SD 14; range 46-81) minutes and 68 (SD 17; range 50-94) minutes, respectively. Clinicians were interviewed across 6 groups (2-4 participants each), lasting 68 minutes on average (SD 7; range 62-80 minutes). Caregivers participated in 1 of 4 focus groups (2-3 participants per group; mean duration of 84 minutes, SD 17; range 69-106 minutes), with one taking part in a qualitative interview (40 minutes).

**Table 3:** Demographic and medical characteristics of participants (primary caregivers).

Primary caregivers		Step 2 (N=11)
Female, n (%)		11 (100)
White, non-Hispanic, n (%)		9 (82)
White, Hispanic, n (%)		2 (18)
**Marital status, n (%)**		
	Single or never married	0 (0)
	With a partner	0 (0)
	Married	10 (91)
	Widowed	0 (0)
	Separated	0 (0)
	Divorced	1 (9)
**Education, n (%)**		
	Some high school or less	0 (0)
	High school diploma or certificate equivalent	1 (9)
	Vocational or trade school	0 (0)
	Some college	1 (9)
	Associate degree	0 (0)
	College degree (eg, BA, BS)	2 (18)
	Graduate or professional degree	7 (64)
**Work or school status, n (%)^a^**		
	Attending school full time	0 (0)
	Attending school part time	0 (0)
	Working full-time	5 (45)
	Working part-time	3 (27)
	Full-time homemaker	4 (36)
	Volunteer full-time	0 (0)
	Volunteer part-time	1 (9)
	Unemployed, seeking work	0 (0)
	Not attending school or employed due to my child’s health	1 (9)
	Not attending school or employed due to my health	0 (0)
	Not attending school or employed due to other reasons	0 (0)

^a^Work or school status item offers “check all that apply” as a response.

#### Thematic Results

##### Overview

Results yielded 3 major themes: (1) intervention structure, (2) intervention materials, and (3) specific session feedback. Tables S3 and S4 in Multimedia Appendices 5 and 6 display sample quotes for subthemes corresponding to the themes for intervention structure and intervention materials, which also are summarized below. Table S5 in Multimedia Appendix 7 reviews the data obtained for specific session feedback. All results were used to subsequently refine the telecoaching intervention.

##### Intervention Structure

Most AYA reported favorably on their overall perception of the intervention, stating that they thought it was good, unique, structured well, etc. Some young adults noted that the coaching aspect would be supportive in different ways (eg, serve as a reminder) and that the intervention could potentially have a positive, and even transformative, impact on some people with CF. A few adolescents noted concerns that it might be a lot to do, however, and some young adults felt that the program would not be something that they would need or want. Clinicians made some practical recommendations. For example, clinicians noted that if financial concerns or problems using treatment equipment arose as a concern for the participant, the coach would have to ensure that the participant reached out to their care team for this sort of guidance. Clinicians also emphasized the importance of having “mock” sessions as part of coach training. Some clinicians noted that it will be helpful to have the additional support of the coach reinforcing similar discussions that other clinicians are having around self-management during patient encounters. Caregivers were highly mixed in their perspectives. Some felt less enthusiastic about the intervention because they thought it would be difficult for their adolescent to find time for telecoaching sessions (in addition to existing CF cares) or that their child would not be interested or committed to finishing it. Other caregivers reported that they could see possible benefits and that it was worth trying. Some suggestions were offered by caregivers including perhaps starting younger (before teen years) with patients, offering an introductory session for parents to feel connected, and sharing intervention content with caregivers (eg, as “touch points”) so that they can discuss with their child and reinforce their child’s efforts.

Regarding session length, most AYA felt that 30 minutes was sufficient time—not too short and not too long. Clinicians generally felt that the half-hour time frame was good, but some recognized that the length of the session might also need to be responsive to the extent of barriers the participant experiences. Caregivers had mixed views—some reported that it was too long, while others thought it was what would be needed, and others suggested having some flexibility to go shorter or longer, as needed. In terms of frequency of sessions, adolescents noted that having 2 weeks between sessions was sufficient for completing tasks and strikes a nice balance between keeping participants engaged but not overwhelming them. Some young adults reported that the frequency was good, while others suggested that once a month might be more reasonable. Clinician and caregiver perspectives aligned well with adolescents, feeling that 2 weeks between sessions keep individuals engaged in the intervention (eg, fosters routine check-ins). AYA reported that scheduling sessions could be challenging, given school or work, activities, and holidays. Many indicated that sessions would need to take place in the evenings or on weekends to be feasible. Caregivers consistently reported a need to use evenings and weekends as well. One caregiver suggested that having a telecoaching session during vest airway clearance would be ideal. Only a few AYA mentioned that day times (eg, early mornings) would be possible. Clinicians consistently recognized that patients likely would prefer evenings and, perhaps more rarely, early mornings; however, they also noted that it would be difficult for coaches to work after-hours if their time is not protected for that schedule. Furthermore, some clinicians emphasized the challenge of putting in long workdays and then having to find the motivation to engage in a telecoaching session in the evening. Nevertheless, many clinicians stated that there could be ways to find some flexibility (eg, looking at their schedules in advance and choosing to stay later if the clinical day is less busy) to address the scheduling challenge. It also was noted that if these services could be billable, it would make flexible scheduling more feasible.

With respect to the overall intervention length, several AYAs indicated that less than 6-7 months would be preferable, but others felt it was a good length to acquire skills and see how they work. Clinicians, for the most part, felt that the intervention length might be too long and could be a deterrent to those who do not want to make that sort of commitment or who might already have low motivation as part of their self-management concerns. Most caregivers felt that the intervention length was appropriate, noting that it would go by fast, and that extended time is needed to build habits; though, some caregivers remarked that it may seem too long. Overall, we obtained mixed views on the proposed length of the telecoaching intervention.

Clinicians and caregivers were asked about their views on who should serve as coach. Clinicians generally reported feeling comfortable serving as a possible coach in this intervention. They felt that the sessions would be feasible to implement with participants and that their preexisting relationship with the patient would likely be an asset to the process. Furthermore, clinicians reported positive views of the proposed monthly supervision meetings, stating that these meetings will provide coaches with feedback and support. Caregivers mentioned that the quality of the coach is essential, with rapport and empathy as central to fostering a good relationship with the participant.

Caregivers specifically were also asked about their potential involvement in the intervention. Most noted that they wanted to at least be aware of what was happening with the intervention, while others stated that such awareness could facilitate their supporting their AYA with skills. Even if not extensive, it was felt that parents being involved were consistent with the overall care approach with CF—that being “teams” working together.

##### Intervention Materials

Given the importance of the intervention binder as a resource for AYA, participants were queried for their perspectives and feedback on it. Generally, opinions on binder format—printed versus online materials—were highly mixed, but some participants recognized that having both options likely is ideal for meeting anyone’s preference. Consistently, AYA and clinicians also reported that the binder, as an intervention tool, and its contents were accessible and helpful. Many caregivers noted that the binder could be particularly useful for parents to stay informed about the intervention, though other caregivers indicated that their child may not use it, especially after the intervention ends. AYA offered a few suggestions for adding to the binder. These included additional resources that participants could access if interested in more information on a topic, as well as contact information and a brief biography (eg, name, hobbies) on their coach so that the participant can get to know them. Furthermore, it was suggested that a chart would be helpful—documenting treatment plans and intervention activities—to keep things organized. Caregivers further felt that including some additional resources (eg, blog sites and websites) would be helpful.

##### Specific Session Feedback

AYA and clinician feedback on specific sessions within the intervention (eg, overall perception; specific considerations for session activities and worksheets) is reviewed in Table S5 in Multimedia Appendix 7. Overall, perceptions were positive. Participants provided their overall perception but also shared some very helpful recommendations to consider when refining session content and materials.

## Discussion

### Principal Findings

The results of this 2-step series of focus groups and qualitative interviews with the same cohort demonstrate the perceived feasibility of telecoaching as a practical approach through a video calling interface, to navigate personalized efforts in improving treatment self-management for AYA with CF. After formulating the intervention based on Step 1 interviews, qualitative data from Step 2 reflected a general acceptance of the community partner-informed, telecoaching intervention formulated for future testing. Broadly, the findings from these focus groups and individual interviews provided diverse input to inform and optimize a telecoaching intervention that teaches care team members to address problems in people with CF managing their complex treatment regimens. Community partner input showed a sensitivity to the diversity of technological access across people with CF, including a potential lack of device and internet access, which we observed to be uncommon yet remains an important consideration. Input also included practical considerations of the timing and frequency of calls, privacy policies, and relevant clinician concerns (eg, care team schedules and fatigue). Notably, AYA concerns regarding possible reduced motivation in the context of a remote video call should be considered when evaluating the impact of telecoaching in future research. Finally, scheduling concerns were a prominent theme across informants, with comments specific to challenges in finding time to dedicate to regular sessions, as well as conflicting schedule preferences between care team members (likely prefer work hours) and AYA (likely prefer evenings and weekends). Consequently, flexibility in scheduling will need to be an important consideration when implementing the telecoaching intervention.

### Strengths and Limitations

Obtaining community partner input when devising a behavioral intervention is an optimal practice; consequently, our methodological approach is a strength. Individuals with lived experience in having to self-manage CF care on a daily basis (ie, patients) or provide tangible support to individuals managing their CF (ie, caregivers and providers) have key perspectives to share regarding what is feasible, acceptable, and useful to include in a behavioral intervention targeting self-management. They are intimately aware of what areas of self-management are challenging and why, and this information is critical when devising the content and structure of a telecoaching intervention. Furthermore, our 2-phase approach included obtaining community partner perspectives in creating the intervention, as well as critical feedback to help us refine what was initially developed. Confirmability and credibility were enhanced by having the same individuals participate in both Step 1 and Step 2 interviews, thereby providing additional opportunities for feedback. Finally, dependability was assured through an audit trail of detailed notes from coding discussions and decisions, all accessible to the coders throughout the project.

Though these findings provide rich detail and context for finalizing our telecoaching intervention content and structure, and in planning for its overall implementation in a clinical trial, our results also have some limitations. First, although participants were recruited from multiple CF care centers, each different in size and region of the United States, there may be some concerns regarding the transferability of study findings. Our AYA and caregiver sample was primarily White and non-Hispanic. Although these demographics are characteristic of much of the CF population (ie, 90.9% of the CF population in 2023 identified as White [7]), our findings may not capture important perspectives and experiences of individuals with CF who come from minoritized backgrounds. Similarly, our CF clinicians were all White and non-Hispanic, which likely does not reflect the demographic distribution for care team members across the United States. In addition, all caregivers and most patients and clinicians identified as female. As the telecoaching intervention continues to be evaluated and implemented, sensitivity to diversity factors will be critical in ensuring that the intervention is relevant and applicable across CF populations.

Second, key historical events arose following the completion of our focus groups. Although these events did not impact our qualitative data, they still should be considered as we move forward with our intervention. The first historical event was the United States Food and Drug Administration’s approval of the Elexacaftor, Tezacaftor, and Ivacaftor combination (ETI) in October 2019, for people with CF aged 12 years and older with at least one F508del mutation. This was a landmark event in the history of treatments for people with CF, given the profound positive health impact of ETI. Indeed, the advent of ETI as a highly effective therapy for the majority of the US CF population spurned further research on the need for continuing multiple airway-clearing treatments in CF (eg, SIMPLIFY clinical trial) [27]. This factor alone shifted treatment regimens (and complexity) for many people with CF as self-driven or care team-informed decision-making began to decrease the number of treatments for some people with CF. For others, the improvements in lung and overall health positively shifted treatment self-management due to increased motivation and energy. This highly effective CFTR modulator has had marked impacts on CF quality of life [28,29]; the associated impact on the overall prescribed treatment regimen and self-management remains an important point of future investigation—one that will clearly be relevant to the implementation and use of our telecoaching intervention.

The second historical event was the COVID-19 pandemic that began in November 2019 and rapidly changed care practices in outpatient US health care delivery, including CF, to use telehealth visits. To protect people with CF who are vulnerable to the spread of respiratory pathogens (including SARS-CoV-2), many CF centers adopted telehealth visits to provide safe access to continued outpatient care. Care team members familiarity with telehealth thus vastly increased in almost all medical fields. Furthermore, patient and family familiarity with the use of video-conferencing technology also increased rapidly across health care, work, and social contexts. The feasibility of videoconferencing for patients and families with CF for use in telecoaching will likely be enhanced given experiences with teleconferencing as a mainstay of communication during the pandemic. Nevertheless, the impact of the COVID-19 pandemic on telehealth services and delivery remains in evolution. Reimbursement for telehealth visits and adjusting licensure for providing telehealth across expanding geographic areas are just two aspects of how the behavioral health field has incorporated the use of teleconferencing to optimize health care delivery within multidisciplinary health care teams. Findings on the feasibility or acceptability of telecoaching, which may closely mirror some aspects of mental health care to lay persons, may be improved after the widespread use of these technologies during the COVID-19 pandemic.

### Future Directions

Telecoaching is gaining applications in the treatment of chronic disease in many areas but remains nascent in CF. To our knowledge, this is the first study in CF to explore and describe the integrated perspectives of patients, family members, and health care clinicians on telecoaching as an intervention in CF to improve treatment self-management. The results of this study informed the structure and content of the telecoaching intervention, which recently was implemented in a feasibility pilot investigation addressing treatment self-management in AYA with CF [30]. In addition, an ongoing European multicenter trial of people with CF aged 12 years and older is integrating telemedicine along with telecoaching to address treatment self-management [31]. This investigation will evaluate the impact of these approaches on CF health outcomes, measuring a primary outcome of time to pulmonary exacerbation [31] while additionally studying impacts on treatment self-management and other features of CF health. The findings of studies such as these will become foundational knowledge for future health care practices to promote disease self-management in CF. In other chronic muco-obstructive disease processes, such as chronic obstructive pulmonary disease, telecoaching has already shown feasibility and acceptability for both patients and coaches in a 3-month intervention to improve physical activity [32]. Usage of the telemonitoring (a step counter) was excellent, although engagement with smartphone tasks was overall lower and decreased with time [32]. The phenomenon of initial uptake followed by declining use of any new technology is not unique. These types of trends may, in fact, support the importance of integrating interactive and interpersonal exchange, like telecoaching, in concert with the use of new technologies to improve treatment self-management significantly and sustainably.

### Conclusions

The results of this 2-part series of focus groups support that the CF community is interested in applying the technology of video conferencing with an interactive coaching intervention as a method to address the challenges of chronic treatment self-management and self-management in CF. While people with CF, family members, and health care clinicians voice unique considerations that are valuable in informing a telecoaching intervention for the CF community, the overall enthusiasm reflected for video calling as part of CF care is an important factor when developing future care models in CF. These findings, which were established in a pre-pandemic era of CF, will be of both contemporary and historic value when studying the feasibility and acceptability of telecoaching and remote monitoring of treatment self-management in a post-pandemic landscape of CF treatment.

## Appendix

Multimedia Appendix 1 Step 1 focus group and qualitative interview guides.

Multimedia Appendix 2 Step 2 focus group and qualitative interview guides.

Multimedia Appendix 3 Table S1: Step 1 video call experience theme and subthemes.

Multimedia Appendix 4 Table S2: Step 1 logistics and content of telecoaching intervention theme and subthemes.

Multimedia Appendix 5 Table S3: Step 2 overall intervention structure theme and subtheme.

Multimedia Appendix 6 Table S4: Step 2 intervention materials theme and subthemes.

Multimedia Appendix 7 Table S5: Summary of specific session feedback (across informants).

## Abbreviations

AYA: adolescents and young adults
CF: cystic fibrosis
CFTR: cystic fibrosis transmembrane conductance regulator
ETI: Elexacaftor, Tezacaftor, and Ivacaftor combination

## Data Availability

All data generated or analyzed during this study are included in this published article.
