# Supplementary material for: Using Community Engagement to Create a Telecoaching Intervention to Improve Self-Management in Adolescents and Young Adults With Cystic Fibrosis: Qualitative Study
Source: J Particip Med. 2025 Jan 20;17:e49941. doi: 10.2196/49941 (PMC11791463; doi:10.2196/49941)
Supplement: Multimedia Appendix 1 [file jopm_v17i1e49941_app1.docx]

# Step 1 Focus Group/Qualitative Interview Guides

## Patient Participants' Guide:

1. Describe your ACCESS to a device with Internet capability and video display (e.g., smartphone, tablet computer, laptop or desktop computer)?
   1. *All age groups*: types of devices, where are the devices available to you (home/work/school), frequency of access
   2. *Adolescents only*: level of parental supervision, personal device or shared with other family members, open access or are there limitations?
2. In the past, have you used video-calling for any purpose (reason) of communication? If so, HOW OFTEN and what was the purpose of this communication?
   1. Why did you choose to do a video call for this purpose? How do you think video calls are different/similar to in person communication? Regular phone call? What do you think are some purposes for which you may *NOT* use video calling?
3. How much do you ENJOY using video-calling?
   1. What do you like and not like about it?
4. If you have ever used video-calling, how EASY was it for you to use?
   1. What makes it easy to use? Any challenges?
5. In your experience, how CLEAR is communication during a video-call?
6. How often, if ever, have you used video-calling to communicate with your CF care team?
   1. Do you think other adolescents/teens/young adults would be interested in video calling with CF care team? Why or why not? What do you think may be some challenges to video-calling with CF care team?
   2. *If have used video call with CF care team member:* Please describe the experience. What sorts of topics did you discuss or would you want to discuss in the future? Would you want to video call with a CF care team member again? Why or why not?
   3. *If have NOT used video call with CF care team member*: Would you be interested in video calling with CF care team member? Why or why not? For what topics?
7. To what extent do you feel a video-call would be USEFUL in communicating with your CF care team?
   1. What types of topics would it be useful to talk with CF care team about through a video call? What types of topics would you NOT like to talk about over a video call?
8. How COMFORTABLE do you think you would be using video-calling to discuss concerns with following your CF treatment plan with a “coach” from your CF care team?
   1. Why or why not comfortable? If not, what could be done to make you or other patients feel more comfortable?
9. Would you be open to working with a “coach” outside of your CF center, but someone who is part of CF care elsewhere (i.e., National or regional; mention through the CFF)?
   1. Why or why not? If no, any things we could do to make you feel more comfortable?
10. How OFTEN would you prefer that video-calls occur with your CF care team “coach” to discuss concerns with following your CF treatment plan? Would it be once a week, once every two weeks, or once a month?
    1. Why this particular frequency of video-calls for coaching? For how long do you think a patient should be participating in these video-calls to allow enough time for them to make progress at improving their adherence?
    2. Is there a specific type of care team that you would like as a coach (nurse, PT, doc)? Why?
11. Do you think there should be a time limit set on these coaching calls?
    1. If yes, what should it be and why?
    2. If no time limit, why and what are some suggestions of average time limit so patients and providers can plan accordingly?
12. We are putting together a tele-coaching intervention where a CF team member will serve as a “coach” to help discuss and address any concerns or barriers you might have in following your treatment regimen. These coaching sessions will take place through video-calling. The intervention will last for about 6 months, with video-calls being more frequent at the beginning and less frequent toward the end of the intervention.

How INTERESTED would you be in taking part in this sort of tele-coaching intervention to help improve adherence to your CF regimen?

- 1. Why or why not interested? What types of challenges do you think you or other patients with CF would have with participating? How could we fix these issues?

## Provider Participants' Guide:

1. Describe your ACCESS to a device with Internet capability and video display as a health care provider (e.g., smartphone, tablet computer, laptop or desktop computer)?
   1. Types of devices, space where devices available (private/public), shared device or designated user
2. In the past, have you used video-calling for any purpose of communication (either as health care provider or in your personal life)? If so, HOW OFTEN and what was the purpose of this communication?
   1. Have you ever used video- calling as a health care provider? If so, please describe.
   2. How do you think video calls differ from in person communication or a regular phone call? What do you think are some purposes for which you may or may not want to use video calling as a health care provider?
3. How much do you ENJOY using video-calling?
   1. What do you like and not like about it?
4. If you have ever used video-calling, how EASY was it for you to use?
   1. What makes it easy for you to use? Any challenges?
5. In your experience, how CLEAR is communication during a video-call?
6. How often, if ever, have you used video-calling to communicate with your CF patients?
   1. What are some benefits and challenges to using video-calling for communicating with CF patients? To what extent do you think adolescent/young adult patients would be interested in video calling with a CF care team member?
   2. *If have used video calling with CF patients:* What types of topics did you cover with patients? What did the patient think of the experience?
   3. *If have NOT used video calling with CF patients:* What topics would you be interested in covering during a video-call?
7. To what extent do you feel a video-call would be USEFUL in communicating with your CF patients?
   1. Why or why not?
8. How COMFORTABLE do you think you would be using video-calling to discuss adherence concerns as a “coach” with a CF patient of yours?
   1. How comfortable do you think patients would be with this?
9. How OFTEN would you prefer that video-call coach sessions occur with your CF patient to discuss adherence concerns? Would it be once a week, once every two weeks, or once a month?
   1. Why would you prefer this particular frequency of video-calls for coaching?
10. Do you think there should be a time limit set on these coaching calls?
    1. If so, what should it be and why?
11. Would it be feasible to have a few patients to coach in your typical weekly schedule? If not, what specific concerns do you have with available time or scheduling logistics?
    1. Is there a specific type of care team that you think would work best as a coach for discussing adherence concerns? Why?
12. We are putting together a tele-coaching intervention where a CF team member will serve as a “coach” to help discuss and address any concerns or barriers a CF patient might have in following his or her treatment regimen. These coaching sessions will take place through video-calling. The intervention will last for about 6 months, with video-calls being more frequent at the beginning and less frequent toward the end of the intervention.

How INTERESTED would you be in taking part in this sort of tele-coaching intervention to help improve adherence with your CF patients?

- 1. Why or why not interested? What are some possible barriers and how could we overcome these to have you participate?
  2. The plan is to implement this intervention as part of a clinical trial to determine if it is effective in changing behaviors for medication adherence. Over what period of time would you be willing to trust that this intervention was actually effective for improving medication adherence? Over what period of time do you think we would need to implement this to see progress for the average patient?
  3. What other training/materials would you want as a “coach” for adherence if we were to provide you scripts, checklists, patient handouts, and tables to help guide what approach to take?
