# Supplementary material for: Using Community Engagement to Create a Telecoaching Intervention to Improve Self-Management in Adolescents and Young Adults With Cystic Fibrosis: Qualitative Study
Source: J Particip Med. 2025 Jan 20;17:e49941. doi: 10.2196/49941 (PMC11791463; doi:10.2196/49941)
Supplement: Multimedia Appendix 2 [file jopm_v17i1e49941_app2.docx]

# Step 2 Focus Group/Qualitative Interview Guides

## Patient Participants' Guide:

1. To get us started, we have given you a SUMMARY of the telecoaching program, which describes how long it will last, how often the coaching sessions will take place, and how long each session will be. And much of this structure is based off feedback we got from people during the first focus group.

**As a person with CF, what are your thoughts about this plan for the STRUCTURE of the telecoaching program?** What are some positive things about it? What are some concerns you might have?

1. We also provided you with a chart, which gives you an OVERVIEW of how the sessions might take place for an example participant with CF, and what skills would be discussed. We want this table to show the parts of the program that will be the same for all participants, as well as the parts that might be different for each participant depending on their individual treatment goals.

**How well does this table show how the telecoaching program works the same, yet differently, for different participants?** How can we make this clearer? What can be added/changed?

1. The first session is an INTRODUCTION TO TELECOACHING and the summary of this session is on pg.3.

**What are your thoughts about this session?** What do you like or dislike about it?

1. The second skill session also is one that all participants will get, and it covers SMART GOALS.

**What are your thoughts about this session?** What do you like or dislike about it?

**Do you have any questions about what we’ve covered so far?**

So now we will get to the skill sessions. For each skill, there is an initial session and a follow-up session which can be repeated as many times as needed to help the participant reach their goal, and they can be used in any order decided by the coach and participant. These next sessions are important and I want to make sure to get everyone’s thoughts on the content of each.

For each session, I will give you a brief summary and then ask for your feedback. We are looking for feedback on what you think is helpful/unhelpful about the session, what you think are positive or negative (good or bad) parts of the session, and what you like or dislike about the session.

1. One of the skill sessions is called PROBLEM SOLVING. Problem solving skills are used to clearly define the problems that get in the way of doing treatments, and then to brainstorm and identify ways to better handle those problems.

**What are your thoughts about this session?** What do you like or dislike about it?

1. Another skill session is called BEHAVIORAL ACTIVATION. It means learning how to do things that help you experience more positive emotions and energy, which can make it easier to do things you have to do – like CF care.

**What are your thoughts about this session?** What do you like or dislike about it?

1. One of the skill sessions deals with STRESS REDUCTION. Stress reduction means learning to identify stress reactions as well as more effective ways to cope with stress.

**What are your thoughts about this session?** What do you like or dislike about it?

1. One of the skill sessions is called BEHAVIORAL STRATEGIES. This means learning various skills to be more aware of behaviors and ways to help get things done that need to be done more regularly.

**What are your thoughts about this session?** What do you like or dislike about it?

1. Another skill session covers COMMUNICATION. Learning communication skills means learning about different ways we communicate and how we can talk with others in our lives (family, friends, care providers) to express our feelings effectively and get questions answered.

**What are your thoughts about this session?** What do you like or dislike about it?

1. Another skill session covers SOCIAL SUPPORT. Social support means the amount and type of support people feel from others in their lives, and the positive impact it has.

**What are your thoughts about this session?** What do you like or dislike about it?

1. A subject of another skill session is MOTIVATION. Motivation is how willing someone is to do something. Sometimes people feel very motivated to work on something, and other times people do not want to change.

**What are your thoughts about this session?** What do you like or dislike about it?

1. One of the skill sessions is called BELIEFS ABOUT HEALTH. This means, the way people think about their health or how they think about CF can impact their behaviors and/or choices.

**What are your thoughts about this session?** What do you like or dislike about it?

1. Almost all sessions have some sort of ACTIVITY/worksheet that the participant will be asked to do between sessions. These activities are intended to help the participant learn the skill being taught.

1. **What do you think about this?**
2. **Can you think of any challenges that might come up with asking people to complete an activity between sessions?**
3. **How could we prevent or work through those challenges?**
4. Our plan includes giving all participants a PROGRAM BINDER at the beginning of the study. This binder will contain printed copies of all materials that will be used during the telecoaching program. There will also be a secure place online where participants can get these materials.
5. **How helpful or unhelpful do you think the binder would be?**
6. **Would you prefer a different method of sharing information?**
7. **Do you have suggestions for anything that should be added or removed from the binder?**
8. If coaches are only available to SCHEDULE video calls during regular work hours (9-5 Mon-Fri), **how well do you think this would work for your schedule or the schedules of other participants?**
9. What times work best if you had to schedule calls outside of 9-5 Mon-Fri? (Early morning, late afternoon, early evening, another time?)
10. Before we WRAP UP, we really value your input on this and want to make sure we have covered everything. …**What else do you want to comment on regarding the telecoaching program, or the content of the skill sessions?** This is a really important question, so I want to be sure that everyone has a chance to share.

## Provider Participants' Guide:

1. First, we’d like to get your perspective and opinions on some BROADER ASPECTS of our intervention. We provided you with a SUMMARY of the OVERALL INTERVENTION STRUCTURE in terms of its duration as well as the frequency and length of sessions.
2. **As a CF healthcare provider, and keeping your center in mind, what are your thoughts regarding this overall plan for the STRUCTURE of the telecoaching intervention?** What are some positive aspects? What are some concerns you might have? Considering specific elements of the intervention (duration, # of sessions, fading of sessions, use of video conference) what stands out in your mind, what feedback do you have?
3. **How flexible/open would you be/do you think coaches would be to having coaching sessions take place outside of standard work hours?** What do you think is feasible? (early morning, late afternoon, early evening, another time?)
4. **(If open to flexible hours) Does your role allow for flexible hours**? (discipline specific) Is this allowed in your work environment? What are the considerations or restrictions on # hours worked per week, billable hours, logistical ramifications?
5. We also provided you with an example INTERVENTION FLOW TABLE depicting how the sessions might take place for an example participant.

**To what extent does this flow table illustrate how the intervention is standard in some ways and tailored in others?** How can we make this clearer? What else should be added/changed?

1. The first session - which all participants receive - serves as an INTRODUCTION TO TELECOACHING and the summary of this session is on pg. 3.

**Is there anything you think should be changed about this session’s approach?** What should be added/changed?

1. The second session is also standard for all participants and covers SMART GOALS. Because SMART goals will be used during all of the telecoaching sessions, we thought it would be important to have a session that clearly covers them at the beginning/outset.

**As a CF healthcare provider, what are your thoughts on this session’s content?**

- 1. What should be changed?
  2. Is there anything that should be added?
  3. Is there anything that should be removed?

**Do you have any questions about what we’ve covered so far?**

For each skill, there is an initial session and a follow-up session which can be repeated as many times as needed to help the participant reach their goal, and they can be used in any order decided by the coach and participant. These next sessions are important and I want to make sure to get everyone’s thoughts on the content of each.

For each session, I will give you a brief summary and then ask for your feedback. We are looking for feedback on what you think is helpful/unhelpful about the session, what you think are positive or negative (good or bad) parts of the session, and what you like or dislike about the session.

1. One of the skill sessions covers PROBLEM SOLVING.

**As a CF healthcare provider, what are your thoughts on this session’s content?** What should be added/changed?

1. Another skill session covers BEHAVIORAL ACTIVATION.

**As a CF healthcare provider, what are your thoughts on this session’s content?** What should be added/changed?

1. One of the intervention skill sessions covers STRESS REDUCTION.

**As a CF healthcare provider, what are your thoughts on this session’s content?** What should be added/changed?

1. One of the skill sessions covers BEHAVIORAL STRATEGIES.

**As a CF healthcare provider, what are your thoughts on this session’s content?** What should be added/changed?

1. Another skill session covers COMMUNICATION SKILLS.

**As a CF healthcare provider, what are your thoughts on this session’s content?** What should be added/changed?

1. Another intervention skill session covers SOCIAL SUPPORT.

**As a CF healthcare provider, what are your thoughts on this session’s content?** What should be added/changed?

1. One of the skill sessions covers MOTIVATIONAL INTERVIEWING.

**As a CF healthcare provider, what are your thoughts on this session’s content?** What should be added/changed?

1. One of the skill sessions covers HEALTH BELIEFS.

**As a CF healthcare provider, what are your thoughts on this session’s content?** What should be added/changed?

1. Our intervention plan includes having coaches and all participants receive a PROGRAM BINDER at the start of the study that contains written copies of all materials that will be used during the intervention. There also will be an online repository of the same materials that can be accessed by coaches and participants.
2. **How helpful or unhelpful do you think the binder would be?**
3. **Would you prefer a different method of sharing information?**
4. **Do you have suggestions for anything that should be added or removed from the binder?**
5. COACH TRAINING will include a live (face-to-face), SMALL GROUP WORKSHOP that will take place over 1 full day or 2 half days. Training will include review of binder materials, extensive role-play activities (experiential practice), Q&A sessions, and a pre-post knowledge/skills assessment. Once coaching begins, all coaches will have monthly supervision with the team’s psychologists via video conference call.

**Given these general TRAINING & SUPERVISION plans, how COMFORTABLE do you think you would be using this intervention to discuss adherence concerns with a patient with CF of yours (if you were a coach)?**

1. Before we WRAP UP, we really value your input on this and want to make sure to go around to everyone so you all have the opportunity to share final feedback. What else do you want to comment on regarding the telecoaching intervention or the content of skill sessions?

## Caregiver Participants' Guide:

1. Before we start reviewing the skill sessions, we’d like to get your perspective and opinions on the BROADER PLANS we have for the study. To get us started, we have given you a SUMMARY of the telecoaching program, which describes how long it will last, how often the coaching sessions will take place, and how long each session will be.

**As a parent or primary caregiver of someone with CF, what are your thoughts about this plan for the STRUCTURE of the telecoaching program?** What are some positive things about it? What are some concerns you might have? Considering specific parts of the program (like how long it lasts, the length and # of sessions, & using video calls), what stands out in your mind / what feedback do you have on these parts of the structure?

1. We also provided you with a chart, which gives you an overview of how the sessions might take place for an example participant with CF, and what skills would be discussed. We want this table to show the parts of the program that will be the same for all participants, as well as the parts that might be different for each participant depending on their individual treatment goals.

**How well does this table explain or show how the telecoaching program works the same, yet differently, for different participants?** How can we make this clearer? What can be added/changed?

Next, we want to give you a brief overview of sessions that will be covered during the telecoaching program.

1. The first session is an INTRODUCTION TO TELECOACHING.

**What are your thoughts about having an introductory session for each participant?** What else do you think we should address in the introduction session? What do you like or dislike about it?

1. The second skill session also is one that all participants will get, and it covers SMART GOALS.

**What are your thoughts about this session?** What do you like or dislike about it?

**Do you have any questions about what we’ve covered so far?**

For each individual skill session, there is an initial session, and as many follow-up sessions as necessary to help the participant reach his or her goal, and they can be used in any order decided by the coach and participant.

For each skill session, we have a brief, one-sentence summary describing the goal of the session to give you a sense about why it may be helpful for an adolescent or young adult with CF who is working on completing their daily treatments. We would like to get your feedback on what you think is helpful/unhelpful about the topics and goals of each session.

1. One of the skill sessions is called PROBLEM SOLVING.

**What are your thoughts about the topic of this skill session?** What do you like or dislike about it?

1. Another skill session is called BEHAVIORAL ACTIVATION.

**What are your thoughts about the topic of this skill session?** What do you like or dislike about it?

1. We also have a skill session that deals with STRESS REDUCTION.

**What are your thoughts about the topic of this skill session?** What do you like or dislike about it?

1. One of the skill sessions is called BEHAVIORAL STRATEGIES.

**What are your thoughts about the topic of this skill session?** What do you like or dislike about it?

1. Another skill session covers COMMUNICATION SKILLS.

**What are your thoughts about the topic of this skill session?** What do you like or dislike about it?

1. We also have a skill session that covers SOCIAL SUPPORT.

**What are your thoughts about the topic of this skill session?** What do you like or dislike about it?

1. The subject of another skill session is MOTIVATION.

**What are your thoughts about the topic of this skill session?** What do you like or dislike about it?

1. One of the skill sessions is called BELIEFS ABOUT HEALTH.

**What are your thoughts about the topic of this skill session?** What do you like or dislike about it?

1. Almost all sessions have some sort of ACTIVITY that the participant will be asked to do between sessions.
2. **What do you think about this?**
3. **Can you think of any challenges that might come up with asking people to complete an activity between sessions?**
4. **How could we prevent or work through those challenges?**
5. Our plan includes giving all participants a PROGRAM BINDER at the beginning of the study. This binder will contain printed copies of all materials that will be used during the telecoaching program. There will also be a secure place online where participants can get these materials.
6. **How helpful or unhelpful do you think the binder would be for participants?**
7. **Would you suggest a different method of sharing information?**
8. **What be added or removed from the binder?**
9. If coaches are only available to SCHEDULE video calls during regular work hours (9-5 Mon-Fri), **how well do you think this would work for the participants**? As a reminder, participants will be ages 14-25 years old.
   1. What times work best if a participant had to schedule calls outside of 9-5 Mon-Fri? (Early morning, late afternoon, early evening, another time?)]
10. Before we WRAP UP, we really value your input as a parent/caregiver, and want to make sure that we include your unique perspectives and feedback when creating this program for AYA with CF. **What are some special considerations we need to give to caregivers when finalizing our Telecoaching program?** This is a really important question, so I want to be sure that everyone has a chance to share.
