# Supplementary material for: Using Community Engagement to Create a Telecoaching Intervention to Improve Self-Management in Adolescents and Young Adults With Cystic Fibrosis: Qualitative Study
Source: J Particip Med. 2025 Jan 20;17:e49941. doi: 10.2196/49941 (PMC11791463; doi:10.2196/49941)
Supplement: Multimedia Appendix 3 [file jopm_v17i1e49941_app3.docx]

## **Table S1**

## **Step 1 Video Call Experience Theme and Sub-Themes**

| **Previous Use of Video Call**  (*Patients Only)* | “I have access pretty much everywhere. I have Internet in my apartment. I'm in college right now, but I do have Internet access within my apartment. And if I have issues there, I can also go on the campus and use the Internet there. So, it's generally not an issue for me.” **(Female patient, 22)**  “I think I might have some parental restrictions, but I'm not entirely sure.” **(Female patient, 17)** |
| --- | --- |
| **Benefits of Video Calls** | “You don't have to go somewhere to see the person first. And then if you want to see, like, their expressions or something like that, see how they react.” **(Female patient, 15)**  "It’s just kind of straightforward, and self-explanatory." **(Female patient, 15)**  “I think it would be beneficial for patients who drive a fair distance, which we have several patients that drive four hours or more to come to clinic. So, if they're having an acute situation and they need to be assessed, I think it would be helpful for them to not have to necessarily make the drive, but we would still be able to visually see them.” **(Clinician, respiratory therapist)** |
| **Challenges of Video Calls** | “If you didn’t have the proper device or WIFI or that kind of thing, could definitely be a challenge if you had to, like, say, go the-- like a Starbucks where they have WIFI or to a library to use a computer, something like that, it could be kind of awkward to be in a Starbucks like, “Oh yeah, got green mucous this morning.” It could be kind of strange.” **(Female patient, 22)**  “There's always technical issues that can occur, even if you have the capabilities on both ends. So that can kind of delay your purpose for using it.” **(Clinician, nurse)**  “The patients having the availability, the access to technology, because we do have some that their circumstances do not allow that.” (**Clinician, nurse practitioner**) |
| **Perceived Patient Interest in Video Call**  *(Clinicians Only)* | “I think it would work really well for all of our age groups at the pediatric center. I think obviously the younger they are, then the parents would fill in for the patient, which is what happens at the appointments anyways. But I think a lot of our parents would take advantage of the video calling.” **(Clinician, respiratory therapist)**  “I can think of patients for sure who would benefit from this. Young adults that are kind of coming on their own, starting to fill their own meds, starting to do things all on their own without the help of their parents, you could really step in and help them out in that way. I mean certainly I think the age group, like 17 to 21 would be great for this.”  **(Clinician, internal medicine/MD)** |
