# Supplementary material for: Using Community Engagement to Create a Telecoaching Intervention to Improve Self-Management in Adolescents and Young Adults With Cystic Fibrosis: Qualitative Study
Source: J Particip Med. 2025 Jan 20;17:e49941. doi: 10.2196/49941 (PMC11791463; doi:10.2196/49941)
Supplement: Multimedia Appendix 4 [file jopm_v17i1e49941_app4.docx]

## **Table S2**

## **Step 1 Logistics & Content of Tele-coaching Intervention Theme and Sub-Themes**

| **Qualification of Coach** | “I can't think of anyone in particular because they all have their own kind of specialty going on. Maybe the nurse just because they know so much about each of the areas in terms of getting everything ready for everybody. So, I feel like they might have like the fullest view of each patient, maybe.” **(Male patient, 23)**  "I would think it would probably be very dependent upon what we're coaching them on. If it's airway clearance or an issue with nebulized medications, it feels like obviously our expert respiratory therapist would be the best. So, I think it would be, to me, it would be very dependent upon what the issue or what the particular thing we want coaching would be." **(Clinician, nurse)** |
| --- | --- |
| **Frequency and Length of Intervention** | “I'd say, I mean once again it's all situational. But like if you're like, your health is down, as you– like if you're doing this and you're getting help and then your health, you start to go up, then it can gradually, the frequency of doing it can go down.” (**Male patient, 16)**  “I would say if they needed weekly coaching that it would probably take at least a month or two, just to kind of—just because it would take that long to get your habits and schedules down and whatnot.” **(Male patient, 24)**  “Since we see our patients, or we're supposed to see our patients quarterly, I would think that we'd want to give it at least three months, three to six months, probably, to see some benefit.” (**Clinician, nurse coordinator**) |
| **Duration of Calls** | “I don't think so, necessarily. Depending if you're doing well, if it’s a 10, 15 minute check in, that's great. Or if you're having issues and you need an hour to kind of work through them and figure out a plan, I think that's good as well.” **(Female patient, 22)**  “Yeah, I feel like if you're going over an hour or even half an hour, you probably should go in. If you have that many concerns that you're like really lamenting on for longer than an hour, you probably should go in clinic and get the full evaluation.” **(Female patient, 24)**  “I think if you mimicked what you're actually doing in clinic– I know for us it's between 20 and 30 minutes per patient. And I think I would try to stick to that and try to stay on the topic, which sometimes can get a little challenging.” (**Clinician, dietician**) |
| **Topics for Calls** | “I think it really depends on the purpose of the video call. I think it would be very nice to be able to call someone on my CF team and be able to talk out options, like I said, if I’m ill and I would like to get better without having to be admitted. I feel like that would be something that would be very nice to be able to talk about. (**Female patient, 17)**  “I think for the purposes of trying to identify their barriers to adherence and helping them overcome those barriers by just providing some insight into them, or if it's a time management issue, trying to brainstorm with them on how they may be able to get that in, a certain type of therapy.” (**Clinician, nurse**) |
| **Clinician Ability to Integrate Tele-coaching**  *(Clinicians Only)* | “It's just how it shakes out, and it's different every time. So, if we were to do coaching, it would probably be outside the hours of clinic or on perhaps a slow– we have one day that's usually a little bit slower where the patients are spaced out more because there's not as many clinicians. But I think it would be an add-on. A different day and time separate from clinic would be the tele-coaching.” (**Clinician, psychologist**) |
| **Clinician Needs to Conduct Tele-coaching**  *(Clinicians Only)* | “The only concern I would have would be availability of finding a room we could actually do a video chat with the patient. That would be my only concern.” (**Clinician, respiratory therapist**)  “If you make it like a fixed time every week it’s like a clinic appointment, the person may need to take off work or go somewhere else to do the call, so it’s making it as easy for both sets of people, not to miss out on the things they are doing to do like a clinic visit that is not really a clinic visit. It’s just like how does the timing work.” (**Clinician, internal medicine/MD)** |
| **Patient Engagement Strategies**  *(Clinicians Only)* | “I think having some of those visual tools can be really– that's not always easy for us to do in clinic, to pull up a computer, to give them graphics or show them those things. So being able to do that in this setting could be beneficial.” (**Clinician, respiratory therapist)**  “Adherence is like, oh, kind of a loaded word, I guess. It's a little more negative than barriers, I would say. We use it all the time, but we don't– when we're talking to patients, we don't necessarily say "are you being adherent with your medication?" And we don't really use the term "what are your barriers?" We just kind of pose questions in a certain way to see what they're doing.” (**Clinician, nurse**) |
