# Supplementary material for: Using Community Engagement to Create a Telecoaching Intervention to Improve Self-Management in Adolescents and Young Adults With Cystic Fibrosis: Qualitative Study
Source: J Particip Med. 2025 Jan 20;17:e49941. doi: 10.2196/49941 (PMC11791463; doi:10.2196/49941)
Supplement: Multimedia Appendix 5 [file jopm_v17i1e49941_app5.docx]

## **Table S3**

## **Step 2 Overall Intervention Structure Theme and Sub-Theme**

| **Overall Impression**  *(All)* | “It’s kind of a lot to do.” (**Female patient, 15**)  “I really think it might help a lot of people, and it looks good.” (**Female patient, 19**)  “If I feel like I'm not getting anywhere with someone, then having somebody else step in and kind of back me, or reiterate what I have discussed with the patient, I think that that can be really helpful.” (**Clinician, respiratory therapist**)  “I think it’s important that the parents or caregivers know what you’re talking about when you’re doing your tele-coaching or the topics that you’re working on. That way you can talk to the kids about it as well.” (**Caregiver**) |
| --- | --- |
| **Session Length**  *(All)* | "The length of time is a great time length to kind of see and get a feel for things." (**Male patient, 22**)  “I think the 30-minute timeframe is good.” (**Clinician, nurse**)  “I can see that it might not necessarily take 30 minutes sometimes, and sometimes it might take more.” (**Caregiver**) |
| **Session Frequency**  *(All)* | “It’s not too overwhelming in the beginning, but it’s still often enough to keep people interested.” (**Female patient, 17**)  “Maybe once a month would be more reasonable.” (**Female patient, 24**)  “It can be hard to keep people engaged and motivated when sessions are monthly or even remember what was discussed in a coaching session that happened 3 or 4 weeks prior.” (**Clinician, psychologist**)  “I think that every other week – every two weeks – gives them something to work on and be able to check in again.” (**Caregiver**) |
| **Session Scheduling**  **Time**  *(All)* | “…weekends and later times [would work], so around 7:00 or 8:00’ish because after school activities or homework or whatever else comes up.” (**Female patient, 15)**  “Early morning or the evening.” (**Male patient, 22**)  "I like to keep my [work] within my schedule. But I also recognize that patients also work, and they might want this after work, too. So, I think it would just depend on whoever's delivering it. Because some people do have that flexibility, so they can do that.” (**Clinician, psychologist**)  “You could do it in the evening during their evening CPT treatments. It’s a 30-minute CPT treatment. You’ve got 30 minutes then.” (**Caregiver**) |
| **Intervention Length**  *(All)* | “…I think that around 4-5 [months] would be preferable.” (**Female patient, 15**)  "I think seven months probably is a good length." (**Female patient, 19**)  “I think people might be a little turned off about the commitment of that many sessions.” (**Clinician, dietitian**)  “You probably need that much time to help them build a habit or a better practice.” (**Caregiver**) |
| **Coaches in the Intervention**  (*Caregivers Only*) | “The quality of the coach makes or breaks the whole program.” (**Caregiver**)  “It needs to be somebody that she identifies with, and she empathizes with and that she feels like empathizes with her.” (**Caregiver**) |
| **Coach Training**  (*Clinicians Only*) | “I think once I get to know the material – most of it is self-explanatory, so I think it would be fairly comfortable or easy to use with the patients.” (**Clinician**, **respiratory therapist**)  “I think it’ll be good to have those [monthly supervision] sessions to help us know the content and what we need to be doing, but I think it’s doable and I think if it’s helpful for our patient, I think we’ll be able to do it.” (**Clinician**, **nurse coordinator**) |
| **Caregiver Involvement** *(Caregivers Only)* | “I think it's important that the parents or the caregivers know what you're talking about when you're doing your tele-coaching or the topics that you're working on. That way [parents] can talk to the kids about it as well.” (**Caregiver**)  “So really, just making sure that the parent is aware that this is something to build on your skills.” (**Caregiver**)  “And also, maybe if I was a parent hearing this, I'd want to know that it's a resource for help.” (**Caregiver**)  “So, if there's a way to make sure it's a team approach, including the parents in some way or aspect, it'd be helpful to caregivers. And that's how we approach clinic. It's a team approach. The families, all the specialists they see. So, to incorporate it that way, I think would make it more inviting.” (**Caregiver**) |
