# Supplementary material for: Using Community Engagement to Create a Telecoaching Intervention to Improve Self-Management in Adolescents and Young Adults With Cystic Fibrosis: Qualitative Study
Source: J Particip Med. 2025 Jan 20;17:e49941. doi: 10.2196/49941 (PMC11791463; doi:10.2196/49941)
Supplement: Multimedia Appendix 6 [file jopm_v17i1e49941_app6.docx]

## **Table S4**

## **Step 2 Intervention Materials Theme and Sub-Themes**

| **Binder Format**  *(All)* | “I’d say that if it was online, it’d be more helpful than on paper.” (**Female patient, 15**)  “The binder, for me, would be better because it’s easy to access and right in front of me.” (**Female patient, 16**)  “I would prefer electronic because it’s always on me and I can access it anywhere…” (**Female patient, 21**)  “Ask whether they want online or binder or both, because sometimes people want both.” (**Female patient, 19**)  “The kids will most likely use whatever you’ve sent them electronically via email to their email account” (**Caregiver**) |
| --- | --- |
| **Binder Usefulness**  *(All)* | “That is super helpful.” (**Female patient, 15**)  “I think it would be super helpful because then you could just have whatever you need right there.” (**Female patient, 22**)  "I think that's a really helpful thing to just have on binder with all your information." (**Female patient, 24**)  “I think the binder’s useful for the parents. The parents should know what’s going on and the parents will use that.” (**Caregiver**) |
| **Suggested Additions to Binder**  *(No Clinicians)* | “A get-to-know-you of your coach…if you don’t know them previously…says their name and hobbies or whatever…that might be helpful just so you can have a better bond with them.” (**Female patient, 15**)  “I think it would be a good idea to have the coach’s contact information in the binder as well.” (**Male patient, 25**)  “A chart would definitely help you to follow along with what you’re doing, your treatment plan and yourself and just keep everything organized.” (**Female patient, 24**)  “What about helpful blog sites or websites where the kids could go to connect with other kids?” (**Caregiver**) |
| **Impression of Session Activities**  *(No Clinicians)* | “Handouts should be a little more variant depending upon the demographic.” (**Female patient, 15**)  "It would be good to have something to keep occupied with what you talked about in your last session and making sure you're still working towards your goal." (**Male patient, 24**)  "I'd hate for it to feel like I'm doing homework every week or two or something like that." (**Male patient, 25**)  "I like having an activity to do in between sessions so that you're not just doing the session and then just forgetting what you did." (**Female patient, 19**) |
