# Supplementary material for: Using Community Engagement to Create a Telecoaching Intervention to Improve Self-Management in Adolescents and Young Adults With Cystic Fibrosis: Qualitative Study
Source: J Particip Med. 2025 Jan 20;17:e49941. doi: 10.2196/49941 (PMC11791463; doi:10.2196/49941)
Supplement: Multimedia Appendix 7 [file jopm_v17i1e49941_app7.docx]

## **Table S5**

## **Summary of Specific Session Feedback (Across Informants)**

| **Module** | **Session Content & Suggested Edits** | **Session Activities & Suggested Edits** |
| --- | --- | --- |
| Introduction | - Gives a sense of what the intervention will be (**Adolescent**) - Getting to know the coach can help improve comfort & build a relationship (**AYA**) - Sets a good tone (**Clinician**) | - Does not require the full session length, so consider combining this session with something else (**Adolescent**) |
| SMART Goals | - Feels like “busy work” and results in “mediocre” outcomes (**Adolescent**) - It is a useful/helpful and different way to set goals, breaking them up, setting timelines, etc. (**AYA**) - Helps to keep someone on track (**Clinician**) | - Recommend doing a couple check-ins and using it to reference at the end of intervention to see how things progressed (**Adolescent**) - Forces someone to think about solutions at hand, and not just barriers (**Clinician**) |
| Problem-Solving | - Good/Helpful because it shows you where to start, walks you through steps, and helps to identify multiple solutions (**AYA**) - It may be difficult to identify solutions for some medications (**Young Adult**) - Need to recognize that other barriers could present problems, even if patient is making progress with the program (**Clinician**) | - Encourage reaching out to others (including coach) in helping to choose a plan (**Adolescent**) - Consider that solutions denoted as “least helpful” might still help (**Adolescent**) - Worksheet is very helpful and clear (**Clinician**) |
| Behavioral Activation | - Would probably use it; want to try it; Good idea (**Adolescent**) - Helpful; helps me plan things; helps me realize and track things that might bring me joy (**Young Adult**) - Confusing about definition & activities; might be a little bit difficult (**Young Adult**) - Can help patients identify times of good and down moods (**Clinician**) | - Incorporate activities to do during treatments; Prefer describing mood rather than using number; Make it bigger for more options (**Adolescent**) - Include weekend category as well; consider using larger blocks of time (i.e., beyond 1-hour) or no set time (**Young Adult**) - Provide example at top; include monitoring for 11:00 PM – 5:00 AM too (**Clinician**) |
| Stress Reduction | - Helpful, specific, & much needed; clarify what type of stress is being talked about (**Adolescent**) - Stress awareness log might be difficult for a typical person to fill out throughout the day (**Young Adult**) - Straightforward and thorough, but could be challenging for some patients (**Clinician**) | - Divide up the day because more than one stressful thing may happen; Simplify/divide into 2 columns to allow tracking in the moment & more detailed response at end of day (**Young Adult**) - Consider adding other relaxation strategies (e.g., progressive muscle relaxation) beyond breathing exercises; clarify more what stress management is and provide examples (**Clinician**) |
| Behavioral Strategies | - Helpful; prizes are good to have (**Adolescent**) - Like that it brings up that it is okay to ask for help; being able to visually see how things are going is helpful (**Young Adult**) - Think that this would be good if patients will use it (**Clinician**) | - Chart is good to check-off things & to see how to fix something (**Adolescent**) - Should be larger; add questions to ask why it is challenging and what could be done better next time (**Adolescent**) - Consider structuring worksheet to consider dosing of medications multiple times a day so that it can be checked off when one might be done; need more than 2 slots for additional treatments/medications (**Young Adult**) - Make things more specific (e.g., divide up types of treatment; separate meals & snacks) (**Clinician**) |
| Communication Skills | - Examples and tips of what to say is useful and makes it more comfortable to talk about it (**AYA**) - Really good set of skills to teach; easy to understand (**Young Adult**) - More helpful session for someone in high school or younger than age 20 (**Young Adult**) - Might be helpful to empower some patients, but also may offend patients who already have these skills (**Clinician**) | - I-messages are not always effective & may place pressure on the patient to share (**Adolescent**) - I-messages get the point across clearly (**Adolescent**) - It is an important tool (**Clinician**) |
| Social Support | - Important, relatable, helps to identify supports (**Adolescent**) - Looks good – nothing wrong with it (**Young Adult**) - Can help the coach learn a lot from the patient (**Clinician**) | - Like it – no changes needed (**Adolescent**) - Diagram is very helpful (**Young Adult**) - Self-explanatory & easy to understand (**Clinician**) |
| Motivation | - Incorporates values & beliefs in a helpful way (**Adolescent**) - Would need to be comfortable to truly share with coach; could easily say motivated, when not (**Adolescent**) - Everything is good (**Young Adult**) - Fairly self-explanatory and hits the highlights of motivational interviewing (**Clinician**) | - Handout and importance scale are helpful (**Adolescent**) - Stressing importance of honesty would help; Consider adding questions about willingness/likelihood in making changes (**Adolescent**) - No changes needed (**Young Adult**) - Great handout with a good scale (**Clinician**) |
| Beliefs About Health | - Really good session; Important; shows the negative effects of not taking their medication (**AYA**) - It may be difficult to convince someone if they feel better despite having stopped medication (**Young Adult**) - Provides the coach with more insight into the patient’s thinking, but also a good tool for self-awareness for the patient (**Clinician**) | - Consider adding extensions to the questions to show the risk of health worsening without change; some people might complete the activity worksheet right before session (**Adolescent**) - Make questions open-ended; Consider adding, “What things could you accomplish if you did your treatments regularly?” (**Young Adult**) |
